# Surface display of PbrR on *Escherichia coli* and evaluation of the bioavailability of lead associated with engineered cells in mice

**DOI:** 10.1038/s41598-018-24134-3

**Published:** 2018-04-09

**Authors:** Changye Hui, Yan Guo, Wen Zhang, Chaoxian Gao, Xueqin Yang, Yuting Chen, Limei Li, Xianqing Huang

**Affiliations:** Department of Pathology & Toxicology, Shenzhen Prevention and Treatment Center for Occupational Disease, Shenzhen, 518020 P.R. China

## Abstract

Human exposure to lead mainly occurs by ingestion of contaminated food, water and soil. Blocking lead uptake in the gastrointestinal tract is a novel prevention strategy. Whole-cell biosorbent for lead was constructed with PbrR genetically engineered on the cell surface of *Escherichia coli* (*E*. *coli*), a predominant strain among intestinal microflora, using lipoprotein (Lpp)-OmpA as the anchoring protein. *In vitro*, the PbrR displayed cells had an enhanced ability for immobilizing toxic lead(II) ions from the external media at both acidic and neutral pH, and exhibited a higher specific adsorption for lead compared to other physiological two valence metal ions. *In vivo*, the persistence of recombinant *E*. *coli* in the murine intestinal tract and the integrity of surface displayed PbrR were confirmed. In addition, oral administration of surface-engineered *E*. *coli* was safe in mice, in which the concentrations of physiological metal ions in blood were not affected. More importantly, lead associated with PbrR-displayed *E*. *coli* was demonstrated to be less bioavailable in the experimental mouse model with exposure to oral lead. This is reflected by significantly lower blood and femur lead concentrations in PbrR-displayed *E*. *coli* groups compared to the control. These results open up the possibility for the removal of toxic metal ions *in vivo* using engineered microorganisms as adsorbents.

## Introduction

Environmental contamination by toxic heavy metals is a serious problem worldwide^1–4^. Lead (Pb), a naturally occurring metal found deep within the ground, has already been redistributed in the environment as a result of human activities over the past thousands of years^5^. Human lead exposure pathways include leaded gasoline^6^, lead-based paints^7^, lead-contaminated food^8^, traditional medicines^9^, the exhaust of diesel fuel^10^, lead containing pipes or lead-based solder in water systems^11,12^, batteries and recycled materials^13,14^. Various measures for public health and environmental protection have been undertaken to decrease lead exposure, such as industrial transformation, the removal of lead from paint, and the introduction of lead free gasoline^6,15,16^. However, due to the non-biodegradable nature of lead, there is lead accumulation in the food chain and persistent lingering lead in the ecological environment^17^. Complete control and prevention of lead exposure is still far from being achieved^2,4^.

Human exposure to inorganic lead occurs mainly through ingestion and inhalation. Lead may be ingested directly from contaminated water, vegetables, fruit, animal meat, and their derivates^18^. Younger children are believed to be most susceptible to environmental lead exposure through oral ingestion of lead exposure and accumulate toxic doses in their bodies^15,16^. Iron, calcium and zinc deficiencies are demonstrated to be associated with increased lead absorption^19^. In addition, dietary factors are usually thought to play an important role in lead intestinal absorption and tissue accumulation. Dietary fibers were shown to be able to bind heavy metals specifically, and some of these fibers possess a high affinity to the lead ions, including pectin, a structural heteropolysaccharide present in the cell wall of terrestrial plants^20^. Oral administration of pectin and its derivatives could decrease lead intestinal absorption and accelerate lead removal in rats^17,21^. Specific adsorbers for lead may inhibit intestinal absorption of lead and are believed to be promising candidates for the prevention and treatment of lead poisoning^22,23^.

Chelation therapy has always been the preferred medical treatment for heavy metal poisoning, but is recommended only for blood lead levels of 45 μg/dL or greater^16^. Existing chelating agents, such as succimer or dimercaprol, have adverse side effects^24^. The safety of chelation therapy for childhood lead poisoning has always been controversial^25,26^. Imbalance of essential elements is very common in the course of chelation treatment, so lead poisoned patients treated with chelating agents need to be monitored closely during and after treatment^27^. Thus, there is a great need for more selective and safer chelating agents for the removal of heavy metal ions^22^.

With the development of molecular display technologies, several heavy metal ion-binding proteins or peptides have become ideal targets for surface display^28,29^. Surface engineered microorganisms as whole-cell adsorbers are considered to be a promising and sustainable approach for the removal of environmental heavy metal pollution^30–32^, and has become one of the research focuses in environmental biotechnology. However, there have been few studies about bioadsorption in biomedical research.

Herein, we have engineered PbrR, a well-known lead specific binding protein from *Cupriavidus metallidurans* CH34^33^, to anchor Lpp-OmpA onto the cell surface of *E*. *coli*, a predominant strain of intestinal commensal bacteria. The aim of the present pilot study is to assess the safety and persistence of surface-engineered bacteria in the murine gastrointestinal tract. The secondary aims are to further evaluate the bioavailability of lead associated with surface-engineered cells and to characterize the amount of lead tissue retention in PbrR displayed *E*. *coli*-treated mice.

## Results

### Construction of surface display vectors

**Figure 1:**
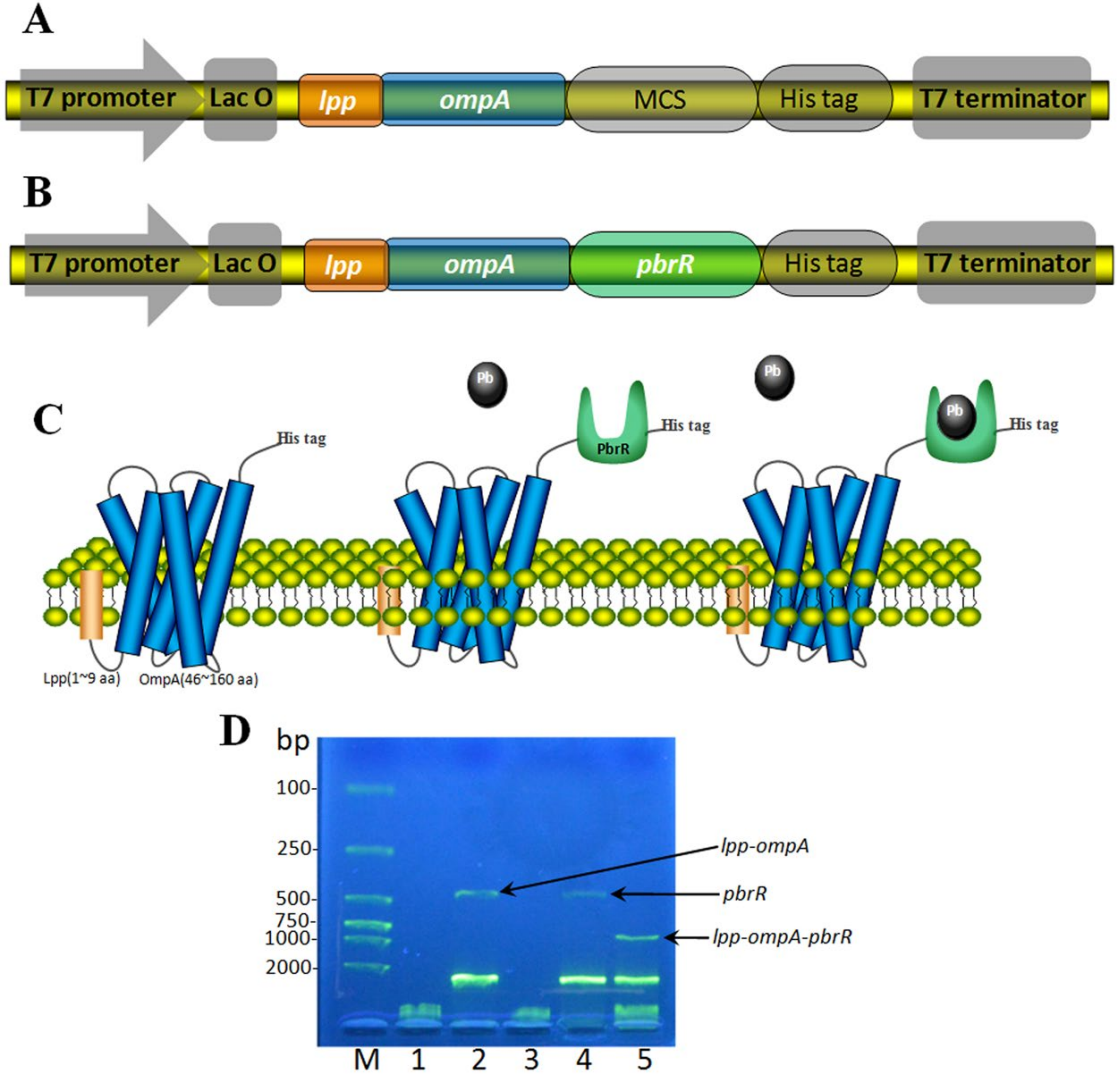
Constructions in this study. Schematic diagrams of pLA (**A**) and pLAP (**B**) cloning/ expression regions and PbrR displayed on the outer membrane of *E*. *coli* (**C**). 2% agarose gel electrophoresis analysis of double restriction enzyme digestion of pLA and pLAP (**D**). Land 1: undigested pLA; Lane 2: pLA digested with *Nde*I and *Sal*I; Lane 3: undigested pLAP; Lane 4: pLAP digested with *Hin*dIII and *Xho*I; Lane 5: pLAP digested with *Nde*I and *Xho*I; Lane M: a low molecular weight DNA marker.

The strategy used in this study to display lead(II)-specific binding protein PbrR on the surface of *E*. *coli* is shown in Fig. 1. Lpp-OmpA is an efficient display system, and it has been successfully used to target a wide range of proteins onto the surface of *E*. *coli* with no obvious adverse effects on cell growth and membrane integrity^34,35^. The cassette encoding surface anchor Lpp-OmpA was first synthesized and inserted into pET-21a to generate pLA for cell surface display of Lpp-OmpA with His tag (Fig. 1A). Expression of anchor Lpp-OmpA-His tag under the control of the T7 promoter/lac operator could be induced by isopropyl β-D-1-thiogalactopyranoside (IPTG)^36^. The cassette encoding PbrR was then synthesized and ligated into the multiple cloning site (MCS) between Lpp-OmpA and His tag encoding sequences in pLA, and the expression vector for the surface display of Lpp-OmpA-PbrR was named pLAP (Fig. 1B). The fusion proteins were tagged with a hexahistidine tag at the C-terminus to facilitate detection with immunological methods. Both of the recombinant vectors were all confirmed by double restriction enzyme digestions, visualization on 2.0% agarose gel (Fig. 1D). An expected band of *lpp-ompA* (approximately 447 bp) was released from pLA by digestion with restriction enzymes *Nde*I and *Sal*I (Fig. 1D, Lane 2). An expected band of *pbrR* (approximately 447 bp) was released from pLAP by digestion with restriction enzymes *Hin*dIII and *Xho*I (Fig. 1D, Lane 4), and the fragment of *lpp-ompA-pbrR* (about 900 bp) was detected after pLAP was digested with restriction enzymes *Nde*I and *Xho*I (Fig. 1D, Lane 5). DNA sequencing analysis was performed to exclude potential mutations introduced by PCR (Fig. S1).

### Surface expression of PbrR using a Lpp-OmpA anchor

**Figure 2:**
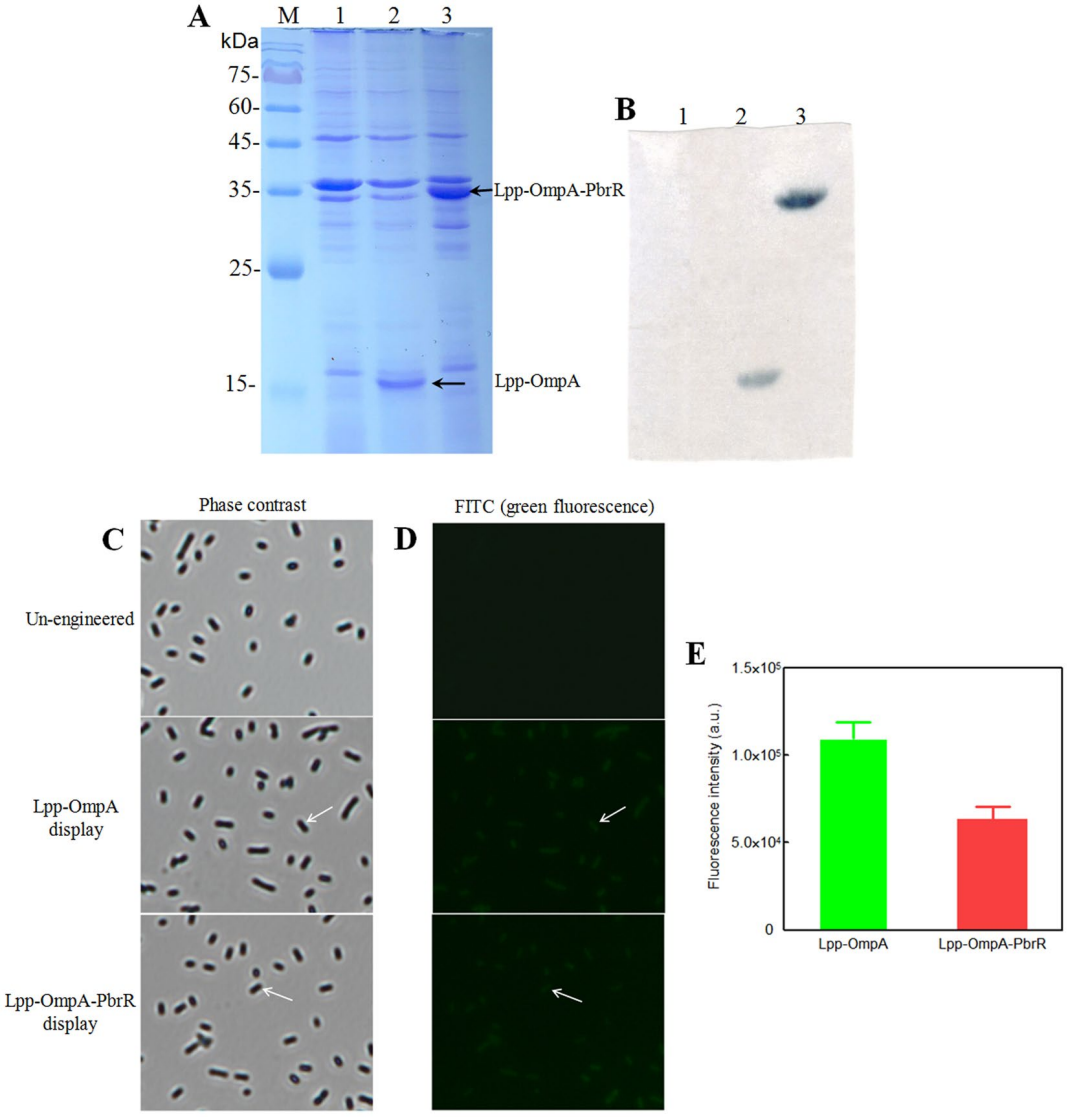
15% SDS-PAGE, immunoblotting and immunofluorescence analysis of surface engineered *E*. *coli*. Total cell proteins were separated by 15% SDS-PAGE (**A**). Western blot of recombinant proteins probed by anti-His antibody (**B**). Lane 1 shows BL21(DE3)pLysS/pLA without induction; Lane 2 shows BL21(DE3)pLysS/pLA after induction (≈17.4 kDa); Lane 3 shows BL21(DE3)pLysS/pLAP after induction (≈33.5 kDa); Lane M is a protein size marker. Phase contrast micrographs (**C**) and immunofluorescence images (**D**) of recombinant *E*. *coli* cells. Immunofluorescence labeling of surface-engineered *E*. *coli* cells using mouse anti-His tag antibody (primary antibody) and FITC labeled donkey anti-mouse IgG antibody (secondary antibody). White arrows indicate individual cells that have been immunofluorescence labeled. Immunofluorescence was performed using a fluorescence microscope (×1000 magnification). Fluorescence intensities of Lpp-OmpA and Lpp-OmpA-PbrR displayed *E*. *coli* cells (**E**) were obtained by subtracting the fluorescence intensity of un-engineered cells. Results shown represent the mean values of three independent experiments.

Denaturing gel electrophoresis of *E*. *coli* high-stringence expression host BL21(DE3)pLysS harboring pLA and pLAP after induction, followed by immunoblotting with anti-His antibody, indicated that Lpp-OmpA and Lpp-OmpA-PbrR were all expressed as full-length products. A protein band of about 17.4 kDa corresponding to the theoretical size of Lpp-OmpA was detected in BL21(DE3)pLysS/pLA after induction (Fig. 2A and B, lane 2). Another protein band of about 33.5 kDa corresponding to the theoretical size of Lpp-OmpA-PbrR was detected in BL21(DE3)pLysS/pLAP after induction (Fig. 2A and B, lane 3).

The surface localization of PbrR was confirmed by immunofluorescence labeling. A hexahistidine tag was added to the C terminus of Lpp-OmpA (pLA) and Lpp-OmpA-PbrR (pLAP) to facilitate detection with an anti-His antibody. Immunofluorescence labeling of *E*. *coli* cells was performed by mouse anti-His IgG as primary antibody, followed by fluorescein isothiocyanate (FITC) labeled donkey anti-mouse IgG as secondary antibody. Signals from FITC (green fluorescence) under the fluorescence microscope with 1000× magnification were observed, and the results are shown in Fig. 2C and D. More than 90% induced *E*. *coli* BL21(DE3)pLysS cells harboring pLA or pLAP showed green fluorescence on their surface. In contrast, no fluorescence was observed on uninduced *E*. *coli* BL21(DE3)pLysS/pLA cells. IPTG induced Lpp-OmpA or Lpp-OmpA-PbrR displayed on *E*. *coli* cell surface was demonstrated.

Furthermore, to evaluate the accessible display of proteins quantitatively, fluorometric assay was also performed after immunofluorescence labeling. The result is shown in Fig. 2E. Lpp-OmpA displayed cells emitted more green fluorescence than Lpp-OmpA-PbrR displayed cells. No extra encoding sequence was inserted into pLA, so the expression and display of a shorter peptide is an energy-saving approach for *E*. *coli* cells. This is consistent with previous studies^37,38^.

### *In vitro* lead(II) adsorption ability of surface engineered cells

**Figure 3:**
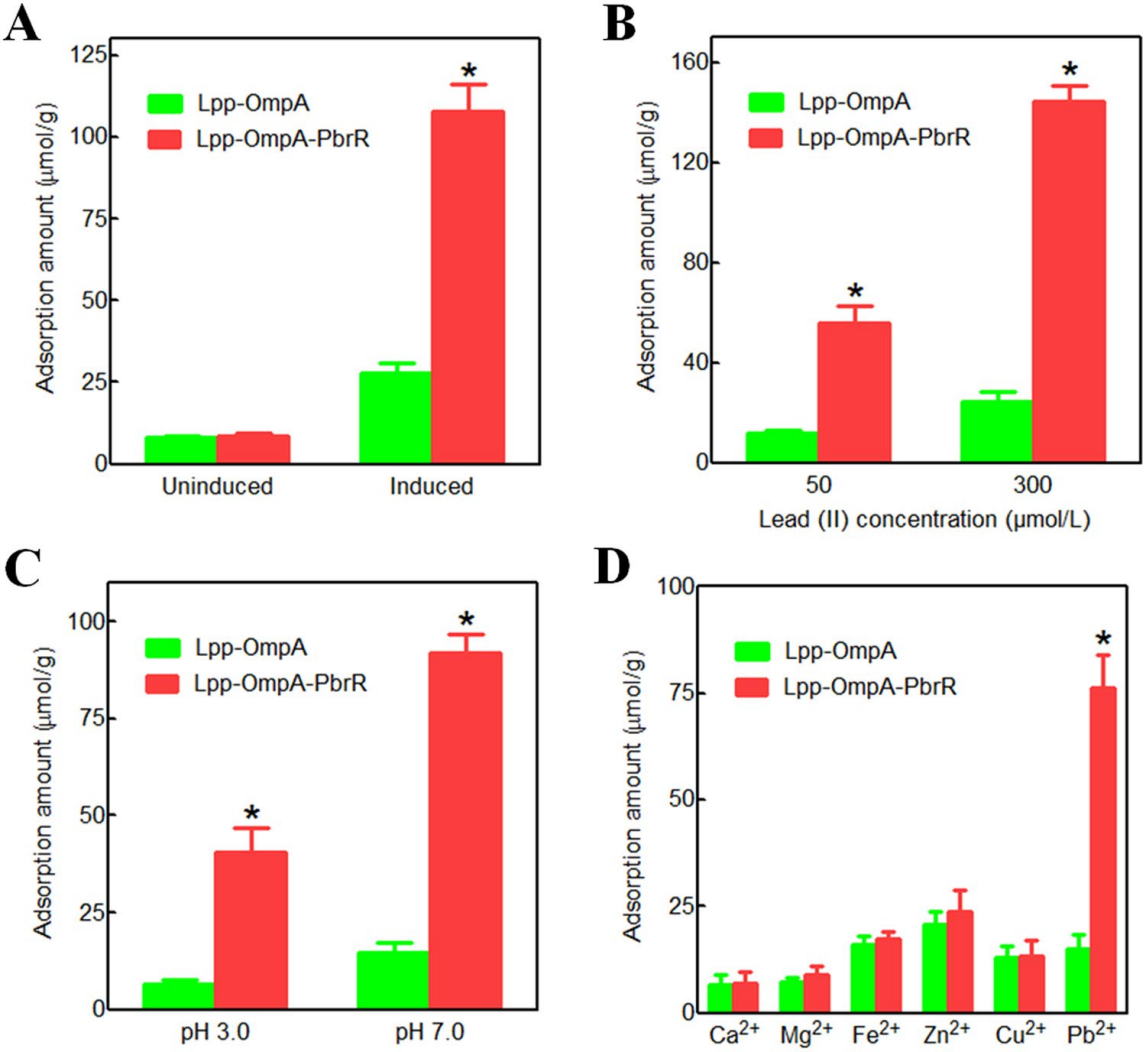
Contribution of surface displayed PbrR to enhanced adsorption of lead(II) *in vitro*. (**A**) Accumulation of lead(II) by cells surface-displayed Lpp-OmpA-PbrR. The adsorption assay was performed in 25 mmol/L HEPES pH 7.0 containing 150 μmol/L lead acetate at 25 °C for 12 h. (**B**) Lead(II) adsorption capacity of cells surface-displayed Lpp-OmpA-PbrR in different concentrations of lead solution. The assay was performed in 25 mmol/L HEPES pH 7.0 containing 50 or 300 μmol/L lead acetate at 25 °C for just 1 h. (**C**) Effect of pH value on lead ion adsorption by cells surface-displayed Lpp-OmpA-PbrR. The assay was performed in 25 mmol/L acetate pH 3.0 or 25 mmol/L HEPES pH 7.0 containing 150 μmol/L lead acetate at 25 °C for 1 h. (**D**) Selective adsorption of lead and other metal elements by cells surface-displayed Lpp-OmpA-PbrR. The assay was performed in 25 mmol/L HEPES pH 7.0 containing 50 μmol/L Pb^2+^, Ca^2+^, Mg^2+^, Fe^2+^, Zn^2+^ and Cu^2+^ at 25 °C for 1 h. The data shown represent the mean values of three independently experiments. *A significant difference (*t test*, *P* < 0.001) between *E*. *coli* surface-displayed Lpp-OmpA-PbrR and *E*. *coli* surface-displayed Lpp-OmpA.

To investigate the capacity of the surface-displayed PbrR to bind lead(II), uninduced or induced BL21(DE3)pLysS/pLA and BL21(DE3)pLysS/pLAP were resuspended in 25 mmol/L HEPES pH 7.0 containing 150 μmol/L lead(II), and the amount of bound lead(II) was quantified by atomic absorption spectroscopy after 12 h. The results are shown in Fig. 3A. *E*. *coli* surface-displayed Lpp-OmpA-PbrR chimera was able to adsorb lead(II) with a capacity of about 108 μmol/g dry cells, which is 4-fold higher than *E*. *coli* surface-displayed Lpp-OmpA (27.7 μmol/g dry cells).

In addition, induced BL21(DE3)pLysS/pLA and BL21(DE3)pLysS/pLAP were incubated with 25 mmol/L HEPES pH 7.0 containing 50 or 300 μmol/L lead(II) for just 1 h. *E*. *coli* surface-displayed Lpp-OmpA-PbrR chimera could adsorb 56 μmol/g dry cells at 50 μmol/L lead(II), and increased to 144 μmol/g dry cells at 300 μmol/L lead(II) (Fig. 3B). Lead adsorption capacity would not increase when the concentration of lead was further increased (data not shown). After the adsorption equilibrium was achieved, the cells above were named unsaturatedly leaded PbrR-displayed *E*. *coli* and saturatedly leaded PbrR-displayed *E*. *coli*, respectively.

To determine the effect of pH value on lead ion adsorption, induced BL21(DE3)pLysS/pLA and BL21(DE3)pLysS/pLAP were incubated with 25 mmol/L acetate pH 3.0 or 25 mmol/L HEPES pH 7.0 containing 150 μmol/L lead(II) for 1 h. *E*. *coli* surface-displayed Lpp-OmpA-PbrR chimera could adsorb 92 μmol/g dry cells at pH 7.0 and 41 μmol/g dry cells at pH 3.0, which were 6.4- and 6.2- fold higher than *E*. *coli* surface-displayed Lpp-OmpA respectively (Fig. 3C).

In order to measure the adsorption selectivity of PbrR-displayed *E*. *coli*, induced BL21(DE3)pLysS/pLA and BL21(DE3)pLysS/pLAP were incubated with a mixed solution containing 50 μmol/L Pb^2+^, Ca^2+^, Mg^2+^, Fe^2+^, Zn^2+^ and Cu^2+^ for 1 h. The results showed that the adsorption of lead ion was significantly higher compared to other major and trace elements (Fig. 3D).

### Oral administration of surface-engineered *E*. *coli* in the short term exerts no adverse effect on mice

In order to determine the effect of oral administration of PbrR-displayed *E*. *coli* on mice, we subsequently performed a short-term *in vivo* toxicity assays using recombinant bacteria^39,40^. No morbidities or mortalities were observed during the experiment as shown in Table 1. Compared with the control group, sustained 15 d oral administration of PbrR-displayed *E*. *coli* at a daily dose of 1 × 10^10^ colony forming unit (CFU) recombinant bacteria exerted no obvious effects on water consumption, on food consumption, on growth rate, and on liver and kidney indices. Furthermore, there was no recombinant *E*. *coli* detected in blood of PbrR-displayed *E*. *coli* treated mice. PbrR-displayed *E*. *coli* did not show any intestinal epithelial invasion nor translocation capability.

**Table 1:** Effect of 15 d PbrR-displayed *E*. *coli* treatment on mice. Liver index calculated as liver weight/body weight ×100%; kidney index calculated as kidney weight/body weight ×100%. Data were presented as mean values ± SEM (n = 10 per group). ND, not determined.

Groups	Water consumption (mL/d)	Food consumption (g/d)	Growth rate (g/d)	Liver index (%)	Kidney index (%)	Bacteria in blood (CFU/mL)
Control	6.24 ± 1.16	4.52 ± 0.85	0.41 ± 0.13	5.51 ± 0.52	1.42 ± 0.15	ND
PbrR-displayed *E*. *coli* treated	6.18 ± 1.24	4.43 ± 0.92	0.39 ± 0.25	5.66 ± 0.41	1.39 ± 0.13	0

### Orally administered surface-engineered *E*. *coli* persists in the murine gastrointestinal tract

**Figure 4:**
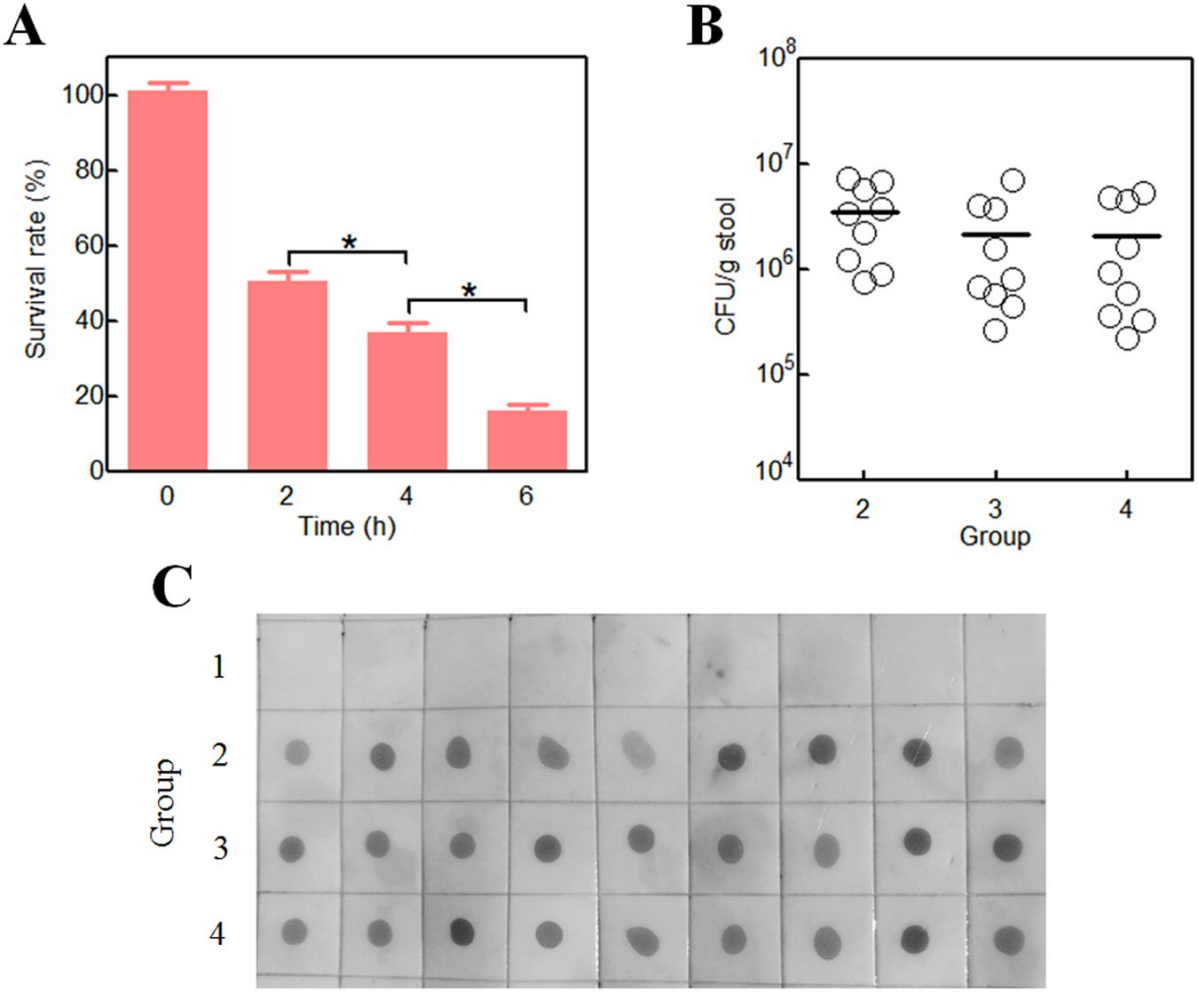
Persistence of surface-engineered *E*. *coli* in the murine gastrointestinal system. Incubation of surface engineered *E*. *coli* in SGJ, and bacterial survival was assayed at certain time intervals (**A**). *A significant difference (One-way ANOVA statistical analysis, *P* < 0.001). Bacterial density in large intestinal contents in mice after receiving Lpp-OmpA displayed *E*. *coli* (group 2), saturatedly leaded PbrR-displayed *E*. *coli* (group 3), unsaturatedly leaded PbrR-displayed *E*. *coli* (group 4) for 15 d (**B**). At day 15, the stool samples in four groups were all obtained. The stool samples in groups 2, 3, and 4 were cultured on LB agar with ampicillin (50 μg/mL) and chloramphenicol (34 μg/mL). Then, the intestinal recombinant bacterial concentration was calculated and indicated as the number of CFU per gram of gut contents (CFU/g stool). Each circle represents one mouse (n = 9), and the bars indicated median CFU values. Stool samples from all four groups were picked. Crude bacterial extracts were separated from the stool samples and dripped on PVDF membrane after cell lysis, and standard Western blot process was done using anti-His IgG as primary antibody to detect the recombinant Lpp-OmpA (Group 2) or Lpp-OmpA-PbrR (Group 3 and 4) (**C**).

The incubation of recombinant bacteria in simulated gastric juice (SGJ) mimicking low pH and highly digestive conditions in the stomach^41^ significantly decreased bacterial survival (Fig. 4A). Half of the added bacteria were alive after a 2 h incubation, and 16% of the bacteria were still alive at 6 h. Using ampicillin resistant (from vector pLA or pLAP) and chloramphenicol resistant (from host *E*. *coli* BL21(DE3)pLysS) properties of recombinant bacteria, the concentrations of these bacteria in stool samples from all three lead-treated groups were determined. There is no significant difference between the three lead-treated groups after 15 d continuous oral administration of recombinant bacteria (Fig. 4B). Furthermore, the integrity of the recombinant protein in stool samples was also tested. A modified Western blot assay based on His tag to detect the full length Lpp-OmpA or Lpp-OmpA-PbrR was established in this study. Dot immunobinding of stool samples with anti-His IgG was performed, and positive results (clear immunospots) were observed in stool samples from all three lead-treated groups with oral administration of recombinant *E*. *coli* (Fig. 4C).

### *In vivo* bioavailability of lead associated with surface-displayed PbrR

To investigate the bioavailability of lead associated with surface engineered *E*. *coli*, the lead ion was supplied as free form (group 2), as saturatedly leaded PbrR-displayed *E*. *coli* (group 3), and as unsaturatedly leaded PbrR-displayed *E*. *coli* (group 4). The final concentrations of lead in semiliquid diet were 1.8 mg lead/kg in all three lead-treated groups. The amount of the semiliquid diet was limited to 100 g/cage.d, so the lead dose was nearly 20 μg lead/mouse.d. Administration of lead took place over 15 days. There was no difference in mouse body weight between the control group and the three lead-treated groups (data not shown).

**Figure 5:**
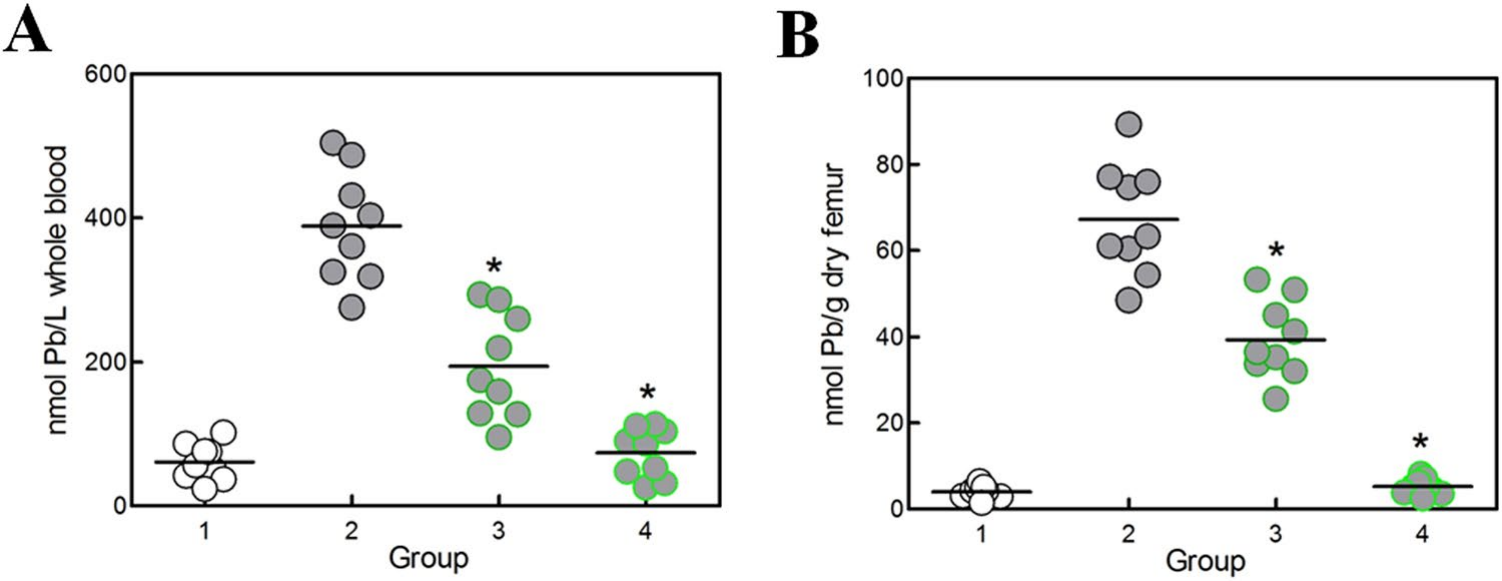
Lead content in whole blood (**A**) and femur (**B**) in mice. Male KM mice were given no lead (group 1), lead acetate + Lpp-OmpA displayed *E*. *coli* (group 2), saturatedly leaded PbrR-displayed *E*. *coli* (group 3), and unsaturatedly leaded PbrR-displayed *E*. *coli* (group 4), in a dose of about 20 μg lead/mouse daily. Blood and femur were harvested on day 15, and lead concentrations were determined. Each circle represents one sample (n = 9), and the bars indicated the mean values. *A significant difference (One-way ANOVA statistical analysis, *P* < 0.001) when compared to group 2, while there is no significant difference between group 1 and group 4.

Lead deposition in the body consists of three major pools: blood, bone, and soft tissues^42^. The primary site of lead storage *in vivo* is bone, and the bone pool (storage pool) may be mobilized and contribute to the blood lead level (exchangeable pool)^42^. Variation in lead storage and mobilization from the storage pool depends on several factors, such as dose/rate of lead exposure, age, pregnancy, and species^43,44^. Lead contents in both blood and bone in all four groups were determined, with the results shown in Fig. 5. As expected, lead treatment in group 2 led to a considerable augmentation in whole blood (>6 fold) and femur (>16 fold) levels of lead, compared to group 1. Interestingly, lead administration in the form of leaded PbrR-displayed *E*. *coli* all led to a significant decrease in both blood and bone lead concentration, compared to group 2. Especially in group 4, in the form of unsaturatedly leaded surface engineered bacteria, lead tissue levels were the lowest among all three lead-treated groups and similar to the control group with no lead exposure.

### Metabolization of physiological metal ions *in vivo* is not affected by oral administration of surface-engineered *E*. *coli*

Major (Ca and Mg) and trace (Fe, Zn, and Cu) element concentrations in blood were measured in all four groups by atomic absorption spectroscopy. The results are shown in Table 2. Due to the highly selective adsorption to lead by PbrR-displayed *E*. *coli*, no obvious difference in five physiological metal ions contents was observed between the control group (group 1) and all three recombinant bacteria treated groups (group 2~4). This is in agreement with the result of the adsorption selectivity of PbrR-displayed *E*. *coli in vitro* as described above.

**Table 2:** Comparison of five physiological metal ion contents in whole blood in all four groups of mice. Data were presented as mean values ± SEM (n = 9 per group).

Groups	Ca (mmol/L)	Mg (mmol/L)	Fe (μmol/L)	Zn (μmol/L)	Cu (μmol/L)
1	3.78 ± 0.38	0.77 ± 0.12	75.01 ± 14.2	22.46 ± 6.15	13.43 ± 7.81
2	3.68 ± 0.41	0.70 ± 0.22	68.35 ± 17.6	20.63 ± 7.88	13.20 ± 6.19
3	3.71 ± 0.29	0.75 ± 0.19	79.22 ± 16.8	21.38 ± 5.35	12.78 ± 8.25
4	3.63 ± 0.52	0.71 ± 0.17	70.14 ± 11.6	20.96 ± 8.23	14.01 ± 8.22

## Discussion

Since the introduction of lead free gasoline worldwide over the last three decades, ingestion has become the primary route of lead exposure, especially for children, due to increased hand-to-mouth activity and enhanced absorption of lead from the digestive tract^16^. Many efforts have recently been made to reduce lead levels in soil, water, and food by focusing on environmental remedies^45–47^. Bioremediation is a waste management technique that involves the use of naturally occurring or genetically modified organisms to detoxify toxic metals by enhancing immobilization and reducing bioavailability of heavy metals^47,48^. The cell surface-display strategy for heavy metal adsorption alleviates the burden of intracellular accumulation, which effectively overcomes the shortcoming of the traditional bioadsorption methods^49^. Heavy metal toxicity in soil or water could be remedied by immobilization of the heavy metal onto the surface of living engineered cells in a non-bioactive form, which was reflected by a positive effect on plant growth and negative effect on toxic metals accumulation in plants^35^. However, there is still no evidence on whether or not surface engineered microorganisms can counteract the toxic effect of heavy metal ions in animals. In this study, a mouse bioassay based on lead oral exposure was performed, in which the bioavailability of lead ion immobilized onto the surface of lead-specific binding protein displayed *E*. *coli* was tested.

The *pbr* resistance operon origin from *Cupriavidus metallidurans* CH34 confers resistance to lead(II) salts, and is regulated by the lead(II) responsive regulator PbrR, which belongs to the MerR family of metal ion-sensing regulatory proteins^33^. The *pbr* operon is the unique lead-specific resistance operon in any bacterial genus so far, and PbrR is the first protein that has been demonstrated to be able to sense lead ions in nature because of its lead-specific binding property^50–53^. By engineering PbrR with the Lpp-OmpA system, we successfully developed whole-cell biosorbent for the selective removal of lead. The production of PbrR on induced *E*. *coli* BL21(DE3)pLysS/pLAP enabled nearly four-fold higher lead(II) biosorption than that found in induced *E*. *coli* BL21(DE3)pLysS/pLA cells (Fig. 3A). Uptake of lead in the digestive tract, from high to low, is via the duodenum, ileum, colon and stomach^16,54^. The binding of metalloregulatory proteins with their recognized metal ions is usually not sensitive to pH value and preferably in slightly acidic pH^32,37,55^. Although stomach is not the main lead uptake site, the extreme acidic environment of gastric juice poses a great challenge to bacterial survival and lead adsorption by PbrR-displayed *E*. *coli*. Compared to the amount of lead adsorption by PbrR-displayed *E*. *coli* at pH 7.0, the amount of lead adsorption was decreased by half at pH 3.0, but it was still 6.2-fold higher than undisplayed *E*. *coli* (Fig. 3C).

The bioavailability of lead(II) bound to the PbrR-displayed *E*. *coli* might primarily depend on the ease of metal release in the gastrointestinal tract. As expected, oral administration of lead(II) immobilized by the PbrR-displayed *E*. *coli* in mice resulted in lower accumulation of lead in both exchangeable pool (blood) and storage pool (femurs) (Fig. 5). Furthermore, unsaturatedly leaded PbrR-displayed *E*. *coli* seemed to provide more free sites for capturing free lead(II) derived from occasional dissociation from the cell surface or from the lysis of engineered cells. It was reflected that tissue lead levels in unsaturatedly leaded PbrR-displayed *E*. *coli* treated group were similar to the control (Fig. 5). More importantly, due to the highly selective lead-binding property of PbrR, there was no difference in the levels of essential mineral cations in the blood between lead-treated groups and the control (Table 2). Lead exposure is known to affect multiple critical organ functions including the nervous, hematological, renal, and cardiac functions^22^. Although lead associated with PbrR-displayed *E*. *coli* was not demonstrated to be orally bioavailable in the current study, its potential detoxifying effect on multiple target organs is worth further investigation in future studies.

Inspired by traditional chelation therapy, researchers have never stopped studying novel oral chelating agents. For instance, low esterified pectin and rhamnogalacturonan-II dimer, molecules rich in free carboxyl groups, have been demonstrated to be ideal specific lead chelators^17,21,56^. They can form strong complexes with lead(II) *in vitro* and lead(II) complexed with them was less bioavailable *in vivo* than lead acetate^56^. Although these kinds of pectic polysaccharides are fermentable and undergo complete fermentation by the gut microflora to liberate the complexed lead^57^, lead absorption occurs primarily in the small intestinal tract and the ileal uptake of lead is very low^58^. Thus, most investigations in animal models have shown a beneficial effect of extra pectic polysaccharides intake on lead detoxification^21,56^.

In order to overcome the shortcomings mentioned above, indigestible biomaterials or metal binding ligands coupled to non-degradable skeletons might be an ideal alternative to pectic polysaccharides. The bacterial cell-surface display strategy for lead adsorption meets the above properties, and has overwhelming advantages over any reported biomaterials, including pectic derivatives, for the following reasons:

First, there are so many microorganisms suitable for host cell-surface display, including some commensal bacteria in the intestines of mammals^28^. *E*. *coli*, a predominant strain among intestinal microflora^59^, was used as the host for surface display of PbrR in this study. The safety and risk assessment of surface-engineered *E*. *coli* was performed in experimental mouse models. Oral administration of surface-engineered *E*. *coli* was demonstrated to have no adverse effect on water and food consumption and growth rate in mice over 15 days and surface-engineered *E*. *coli* had no intestinal epithelial invasion capability *in vivo* (Table 1). The significant decrease of live bacteria during passage of the upper digestive tract is an important drawback for probiotic preparations^60^. The data from our experiment showed that about half of PbrR-displayed *E*. *coli* was killed in SGJ in the first 2 h (Fig. 4A). Since almost no extra lead(II) binding sites exited on the surface of saturatedly leaded PbrR-displayed *E*. *coli*, the degradation of *E*. *coli* in stomach could result in elevated tissue lead content in group 3 (Fig. 5). However, further studies *in vivo* should be done, because the food matrix usually confers extra protection on bacterial survival in the stomach^61^.

Second, abundant colonization in the gastrointestinal tract is one of the most important characteristics of commensal bacteria. Although there was a difference in the oral administration dose of recombinant bacteria, there was no significant difference in recombinant bacterial densities in all three lead-treated groups (Fig. 4B). The stability of the gut microbiota is important for the maintenance of intestinal microflora balance^62^. Besides the survival and persistence of recombinant *E*. *coli*, the integrity of displayed PbrR is another important factor for the immobilization of lead(II) on the surface of recombinant *E*. *coli*. However, PbrR displayed on the outer membrane of *E*. *coli* might easily be hydrolyzed by digestive enzymes in the gastrointestinal tract. An immunoblotting method was established in the study to detect His tag at the C terminus of Lpp-OmpA or Lpp-OmpA-PbrR. The integrity of target protein displayed on the surface was confirmed, but there was a significant difference in the color depth of brown sediments in all three lead-treated groups with oral administration of surface engineered *E*. *coli* (Fig. 4C), and it did not seem to be consistent with similar bacterial densities among three groups (Fig. 4B). Inducible surface display vector derived from pET-21a was used in this study. Although it is suitable for the strict regulation of protein expression in *in vitro* studies, it is impossible to realize the surface display of PbrR during recombinant bacteria survival, growth, and replication in murine intestines. Thus, it might be a good explanation for the results of the *in vivo* study (Fig. 4). To address uncertainties derived from PbrR expression in *in vivo* studies, it would be beneficial to realize the constitutive expression and surface display of PbrR during bacterial colonization.

Finally, the survival and persistence of commensal microflora in the gastrointestinal tract is a unique characteristic that other biomaterials do not possess^63^. More importantly, commensal bacteria always adhere to intestinal epithelium^59^, which is the main uptake site for nutrients including heavy metal ions. Our preliminary results suggested that surface displayed PbrR in commensal bacteria might help block lead uptake continuously. In addition to significantly decreasing blood and tissue lead levels, further studies on lead accumulation, neurotoxicity, and long-term effects are being conducted in our laboratory to determine the *in vivo* feasibility of using biosorption technology to prevent host lead poisoning.

In conclusion, our findings show that lead(II) associated with surface engineered *E*. *coli* is essentially unavailable for intestinal absorption. Engineering the specific toxic metal binding protein on the surface of commensal bacteria provides an alternative method for the prevention of heavy metal poisoning. Growing microbial biomass with the potential for blocking heavy metal uptake *in vivo* needs further investigation. Due to specific and effective binding with lead, surface engineered bacteria, as a kind of probiotic, may be used to decrease lead absorption, prevent lead accumulation, and further ameliorate lead toxicities. However, a series of additional studies in animals are still required before developing surface engineered probiotics as a preventive or perhaps curative agent in lead exposure and toxicity in humans.

## Materials and Methods

### Strains and agents

The strains and vectors used in this study are shown in Table S1. *E*. *coli* Top10 stored in our laboratory was used for all cloning steps. For recombinant proteins expression, pET-21a vector (Novagen, Madison, WI) was used with expression host *E*. *coli* BL21(DE3)pLysS (Novagen, Madison, WI). Restriction enzymes, Pyrobest DNA Ploymerase, dNTP, DNA Marker and gel extraction kits were obtained from TaKaRa (Dalian, China). The mouse anti-His monoclonal antibody and enhanced horseradish peroxidase (HRP)-Diaminobenzidine (DAB) chromogenic substrate kit were obtained from Tiangen (Beijing, China). FITC labeled donkey anti-mouse IgG, HRP labeled rabbit anti-mouse IgG, lead acetate, IPTG, and antibiotics were all purchased from Sangon Biotech (Shanghai, China). Pepsin (1:3000) was obtained from Amresco (OH, USA). Tryptone and yeast extract were obtained from Oxoid (Basingstoke, UK). *E*. *coli* was cultured in Luria-Bertani (LB) broth (1% tryptone, 0.5% yeast extract and 1% NaCl) supplemented with antimicrobial agents, as necessary.

### Construction of recombinant plasmids

DNA encoding surface anchor Lpp-OmpA (LOA), which contains a lipoprotein signal peptide and the first nine N-terminal amino acids of lipoprotein (NCBI Accession No. BAA16044) attached to five outer membrane-spanning domains of OmpA (NCBI Accession No. ABJ00366), was synthesized by ABI 3900 synthesizer in Sangon Biotech Co., Ltd (Shanghai, China). The synthesized fragment (435 bp) was purified by HPLC and directly cloned into pUCm-T using T/A cloning kit (Sangon, Shanghai, China) to generate pT-*loa*. The fragment encoding Lpp-OmpA (447 bp) was amplified from pT-*loa* with primers *Loa-*F and *Loa-*R (Table S2), digested with *Nde*I and *Sal*I and ligated into similarly digested pET-21a to yield pLA. The gene of *Cupriavidus metallidurans* CH34 PbrR (NCBI Accession No. ABF12805) was synthesized according to the codon usage bias of *E*. *coli*, and subcloned into pUCm-T to generate pT-*pbrR*. The fragment encoding PbrR (447 bp) was amplified from pT-*pbrR* with primers *pbrR-*F and *pbrR-*R (Table S2), digested with *Hin*dIII and *Xho*I and ligated into similarly digested pLA to produce pLAP. All recombinant plasmids were confirmed by double restriction enzymes digestion, and finally DNA sequencing was performed using T7 terminator primer (Sangon, Shanghai, China) to confirm accordance with the design.

### Protein expression and analysis

BL21(DE3)pLysS was transformed with pLA or pLAP using CaCl_2_-mediated transformation. To express recombinant fusion protein, overnight cultures of BL21(DE3)pLysS carrying pLA or pLAP were inoculated at 1:100 and grown in LB broth at 37 °C to an optical density at 600 nm of ~0.5, then induced with 0.5 mmol/L IPTG at 25 °C for 12 h. Cells were harvested and resuspened in loading buffer. After boiled for 10 min, samples were run on a 15% SDS-PAGE gel and stained with Coomassie blue dye.

The presence of Lpp-OmpA and Lpp-OmpA-PbrR were confirmed by Western blot using mouse anti-His antibody at a 1:2000 dilution in 20 mmol/L phosphate buffer, pH 7.5 with 0.05% Tween 20 (PBST), and second HRP labeled rabbit anti-mouse IgG, at a final 1:2000 dilution in PBST. HRP signal was detected with enhanced HRP-DAB chromogenic substrate kit.

### Immunofluorescence detection of surface-engineered *E*. *coli* cells

To confirm the presentation of recombinant proteins on the *E*. *coli* outer surface, immunofluorescence labeling using anti His-tag IgG was performed as described below. A 500 μL of uninduced/induced culture was centrifuged at 3500 rpm for 5 min and fixed with 4% formaldehyde in PBS (pH 7.4) for 1.5 h, then blocked in PBS containing 1% fetal calf serum for 30 min prior to immunostaining. The cells were incubated with mouse anti-His antibody diluted 1:200 in PBS overnight at 4 °C. After washing three times with PBS, the cells were then incubated with FITC-conjugated donkey anti-mouse IgG antibody diluted at 1:300 in PBS for 1.5 h at room temperature. After washing three times with PBS, the cells were mounted on slides. Finally, FITC immunofluorescence was visualized using a Nikon Eclipse Ni fluorescence microscope coupled to a Nikon DS-Ri2 digital camera (Tokyo, Japan). The same setting for imaging was applied for all the samples, and representative images were acquired. To evaluate the display efficiency, about 500 cells were counted for each strain, and the percentage of fluorescent cells was defined as (number of fluorescent cells/total cells) ×100%.

### Fluorometric assay

After immunofluorescence labeling, *E*. *coli* cells were suspended in PBS (pH 7.4) and adjusted to an OD_600_ of 1.0. The fluorescence intensity was measured with EnVision 2104 Multilabel Reader (Perkin Elmer, USA) on a 96-well plate. The amount of Lpp-OmpA or Lpp-OmpA-PbrR displayed on the cell surface was quantified by detecting the fluorescence of FITC that attached on the cell surface.

### *In vitro* lead binding experiments

For lead ion adsorption, uninduced or induced cultures of BL21(DE3)pLysS carrying pLA or pLAP were harvested (3500 rpm, 10 min), washed twice with 25 mmol/L HEPES (pH adjusted to 7.0 with NaOH), resuspended to a final OD_600_ of 1.0 in the same buffer containing 150 μmol/L lead acetate and incubated at 25 °C for 12 h with gentle shaking. The cells were collected, washed, dried (80 °C, overnight), weighed and digested with nitric acid. The amount of cells bound lead(II) was finally determined using a Perkin Elmer 600 atomic absorption spectrometer (Waltham, USA).

To investigate the effect of pH value on lead ion adsorption, induced cultures were harvested, washed twice with 25 mmol/L HEPES (pH 7.0) or 25 mmol/L acetate (pH adjusted to 3.0 with HCl), then resuspended to a final OD_600_ of 1.0 in 25 mmol/L HEPES (pH 7.0) or 25 mmol/L acetate (pH 3.0) containing 150 μmol/L lead acetate and incubated at 25 °C for 1 h with gentle shaking. The lead content in the samples was measured by the atomic absorption spectroscopy.

To measure the adsorption selectivity, induced cultures were harvested, washed twice with 25 mmol/L HEPES (pH 7.0), then resuspended to a final OD_600_ of 1.0 in the same buffer containing 50 μmol/L Pb(NO_3_)_2_, Ca(NO_3_)_2_, Mg(NO_3_)_2_, Fe(NO_3_)_2_, Zn(NO_3_)_2_ and Cu(NO_3_)_2_, and incubated at 25 °C for 1 h with gentle shaking. After the treatment, various metal ion contents were all measured by the atomic absorption spectroscopy.

### PbrR-displayed *E*. *coli*/lead complex preparation

Subsequently, induced cultures of BL21(DE3)pLysS/pLAP were harvested, washed, resuspended in 25 mmol/L HEPES (pH 7.0) containing 50 or 300 μmol/L lead acetate, and incubated at 25 °C for 1 h. Then the cells were collected, and extensively washed three times with 25 mmol/L HEPES (pH 7.0). Two groups of *E*. *coli* pellet were named, respectively, unsaturatedly leaded PbrR-displayed *E*. *coli* and saturatedly leaded PbrR-displayed *E*. *coli*.

### *In vivo* toxicity assay of PbrR-displayed *E*. *coli*

*In vivo* toxicity assay of recombinant bacteria was done as described previously^39,40^. Male Kunming (KM) mice, 4~5 weeks old and weighing 18~22 g, obtained from Guangdong Medical Laboratory Animal Center, were used for *in vivo* toxicity assay. All animal experiments were performed in accordance with the guidelines of China Council on Animal Care and Use, and experimental protocols were approved by the Ethics Committee at Shenzhen Prevention and Treatment Center for Occupational Disease. After the adaptation period, twenty KM mice were randomly divided into a control group and PbrR-displayed *E*. *coli* treated group (n = 10 per group). Induced cultures of BL21(DE3)pLysS/pLAP were freshly prepared daily, and the PbrR-displayed *E*. *coli* treated group was orally given at a dose of 1 × 10^10^ CFU/d. The control group was orally given the same volume of saline daily. Control and PbrR-displayed *E*. *coli* treated group were provided with purified water and standard feed ad libitum. During this study, both feed and water consumptions were recorded daily, and body weight increasing rate was calculated. The experiment was performed for 15 d. All mice were killed under anesthesia. Inner organs were removed and weighed. Blood samples in the PbrR-displayed *E*. *coli* treated group were collected, and spread on LB agar with ampicillin (50 μg/mL) and chloramphenicol (34 μg/mL) to detect the presence of recombinant bacteria in blood.

### Simulated gastric juice tolerance of recombinant bacteria

SGJ was prepared using 0.08 mol/L HCl and 0.2% NaCl. The pH value was adjusted to 2.5 using a calibrated pH meter. The SGJ solution was sterilized at 121 °C for 20 min. Pepsin (1:3000) was freshly added at 3 U/mL^41^. Induced BL21(DE3)pLysS/pLAP was incubated in SGJ at 37 °C for 0, 2, 4, 6 h. A 0.2 mL aliquot of the sample was removed at each time point, then the standard serial dilution method of viable bacteria enumeration was done^64^. Bacterial survival rate was calculated as a percentage of the total added bacteria, which was defined as 100%. Experiments were repeated at least three times with similar results.

### Animal experimental design

Male KM mice, 4~5 weeks old and weighing 18~22 g, were used in this study. After the adaptation period, thirty-six KM mice were randomly divided into four groups (n = 9 per group) (Fig. S2). These groups consisted of a control group (group 1) and three experimental groups that received lead as different form (group 2, 3 and 4). Powdered diets (50 g/group/d) were mixed daily with 50 mL of distilled water (group 1), 50 mL of suspension containing 3.6 mg/L lead as lead acetate + Lpp-OmpA displayed *E*. *coli* (group 2), 50 mL of suspension containing 3.6 mg/L lead as saturatedly leaded PbrR-displayed *E*. *coli* (group 3), and 50 mL of suspension containing 3.6 mg/L lead as unsaturatedly leaded PbrR-displayed *E*. *coli* (group 4) to form a semi-liquid mixture that was prepared on site. After 15 days on control or experimental diets, all mice were killed under anesthesia. Subsequently, blood samples (500 μL) were collected into heparinized tubes via the orbital venous plexus, and the left femur was then dissected, frozen at −20 °C until analysis. The stool samples in all groups were obtained from the large intestine, and recombinant bacterial density and recombinant protein immunoblotting analyses were done as described below.

### Assay of recombinant bacterial density and recombinant protein in mice large intestinal contents

A 0.25 g aliquot of fresh stool was homogenized in 0.9 mL aseptic normal saline. Then, one 0.1 mL aliquot was serially diluted and spread on LB agar with ampicillin (50 μg/mL) and chloramphenicol (34 μg/mL) to calculate the recombinant bacterial density in stool sample, and the result was indicated as the number of CFU per gram of gut contents.

The above-mentioned suspension was centrifuged (1000 rpm for 3 min). The supernatant was transferred into another tube and further centrifuged (3500 rpm for 5 min). The supernatant was discarded, and then the pellet was thoroughly washed twice with normal saline (3500 rpm for 5 min). Finally, the pellet was suspended in 40 μL lysis buffer (200 mmol/L Tris-HCl, pH 6.8, 200 mmol/L DTT, 2% SDS), and boiled for 10 min. An aliquot of 5 μL of each sample was dripped on PVDF membrane which was blocked, washed, and incubated with mouse anti-His IgG. After extensive washing, the membrane was incubated with a HRP labeled rabbit anti-mouse IgG, and bound antibodies were visualized using an enhanced HRP-DAB chromogenic substrate kit as mentioned above.

### Determination of lead and physiological metal ions content in biological samples

The metal ion content in blood and femur was determined by the method described previously^56^. Whole blood and femur were dried (80 °C, overnight) and powdered. The powder was dissolved in 0.5 mL of 14 mol/L HNO_3_ and 0.2 mL of 10 mol/L H_2_O_2_ and heated at 140 °C for 2.5 h. An appropriated dilution with purified water was performed in each sample, prior to determination of the concentrations of various metal ions by atomic absorption spectroscopy.

## Electronic supplementary material

Supplementary Information
