# Supplementary material for: Surface display of PbrR on Escherichia coli and evaluation of the bioavailability of lead associated with engineered cells in mice
Source: Sci Rep. 2018 Apr 9;8:5685. doi: 10.1038/s41598-018-24134-3 (PMC5890273; doi:10.1038/s41598-018-24134-3)
Supplement: Supplementary file 1 — Supplementary Information [file 41598_2018_24134_MOESM1_ESM.pdf]

Supplemental Information

Surface display of PbrR on *Escherichia coli* and evaluation of the bioavailability of lead associated with engineered cells in mice

Changye Hui*, Yan Guo, Wen Zhang, Chaoxian Gao, Xueqin Yang, Yuting Chen, Limei Li, & Xianqing Huang

Department of Pathology & Toxicology, Shenzhen Prevention and Treatment Center for Occupational Disease, Shenzhen, 518020 China, P.R.China

Correspondence and requests for materials should be addressed to C.H. (email: hcy_sypu@hotmail.com)

Supplemental Figure

A

The codon optimized DNA sequence of *lpp-ompA*:

ATGAAAGCTACTAAACTGGTACTGGGCGCGGTAATCCTGGGTTCTACTCTGCTGGCAGGTTGC
TCCAGCAACGCTAAAATCGATCAGGGAATTAACCCGTATGTTGGCTTTGAAATGGGTTACGAC
TGGTTAGGTCGTATGCCGTACAAAGGCAGCGTTGAAAACGGTGCATACAAAGCTCAGGGCGT
TCAACTGACCGCTAAACTGGGTTACCCAATCACTGACGACCTGGACATCTACACTCGTCTGGG
TGGCATGGTATGGCGTGCAGACACTAAATCCAACGTTTATGGTAAAAACCACGACACCGGCGT
TTCTCCGGTCTTCGCTGGCGGTGTTGAGTACGCGATCACTCCTGAAATCGCTACCCGTCTGGA
ATACCAGTGGACCAACAACATCGGTGACGCACACACCATCGGCACTCGTCCGGACAAC

Deduced amino acid sequence of Lpp-OmpA:

Lpp signal peptide and 1st 9 a.a.

MKATKLVLGAVILGSTLLAGCSSNAKIDQGINPYVGFEMGYDWLGRMPYKGSVENGAYKAQGV

OmpA a.a. 46 - 160

QLTAKLGYPITDDLDIYTRLGGMVWRADTKSNVYGKNHDTGVSPVFAGGVEYAITPEIATRLEYQ

WTNNIGDAHTIGTRPDNG

The sequencing graph of pLA:
T7 terminator primer was used as the sequencing primer, and the target sequence (435 bp) was highlighted in yellow.

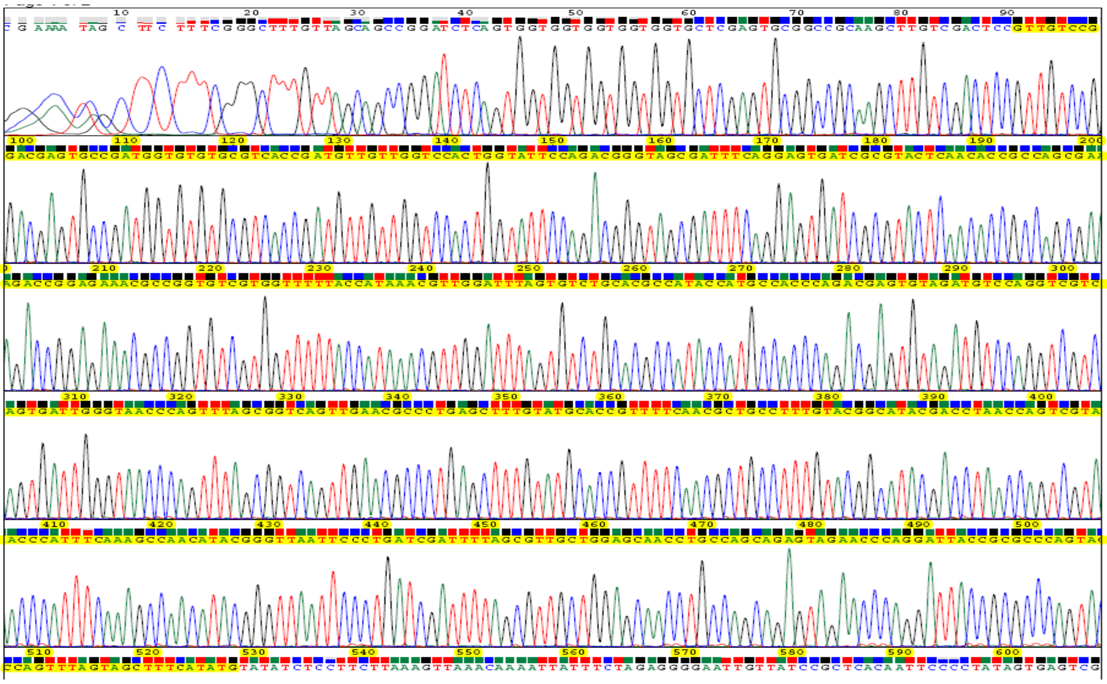

B

The codon optimized DNA sequence of *lpp-ompA-pbrR*:

ATGAAAGCTACTAAACTGGTACTGGGCGCGGTAATCCTGGGTTCTACTCTGCTGGCAGGTTGC
TCCAGCAACGCTAAAATCGATCAGGGAATTAACCCGTATGTTGGCTTTGAAATGGGTTACGAC
TGGTTAGGTCGTATGCCGTACAAAGGCAGCGTTGAAAACGGTGCATACAAAGCTCAGGGCGT
TCAACTGACCGCTAAACTGGGTTACCCAATCACTGACGACCTGGACATCTACACTCGTCTGGG
TGGCATGGTATGGCGTGCAGACACTAAATCCAACGTTTATGGTAAAAACCACGACACCGGCGT
TTCTCCGGTCTTCGCTGGCGGTGTTGAGTACGCGATCACTCCTGAAATCGCTACCCGTCTGGA
ATACCAGTGGACCAACAACATCGGTGACGCACACACCATCGGCACTCGTCCGGACAACGGAG
TCGACAAGCTTATGAATATCCAGATCGGCGAGCTTGCCAAGCGCACCGCATGCCCGGTGGTG
ACCATTCGCTTCTACGAACAAGAAGGGCTGTTGCCGCCGCCGGGCCGCAGCCGGGGGAATT
TTCGCCTGTATGGCGAGGAGCACGTGGAGCGCTTGCAGTTCATTCGTCACTGCCGGTCTCTG
GATATGCCGTTGAGCGACGTACGGACCTTATTGAGTTACCGGAAGCGGCCCGACCAGGATTG
CGGTGAAGTCAATATGCTCTTGGATGAGCACATCCGTCAGGTCGAATCTCGGATCGGAGCCTT
GCTCGAACTGAAGCACCATTTGGTGGAACTGCGCGAAGCCTGTTCTGGTGCCAGGCCCGCCC
AATCGTGCGGGATTCTGCAGGGACTGTCGGACTGCGTGTGTGATACGCGGGGGACCACCGC
CCACCCAAGCGAC

Deduced amino acid sequence of Lpp-OmpA-PbrR:

Lpp signal peptide and 1st 9 a.a.

MKATKLVLGAVILGSTLLAGCSSNAKIDQGINPYVGFEMGYDWLGRMPYKGSVENGAYKAQGV

OmpA a.a. 46 - 160

QLTAKLGYPITDDLDIYTRLGGMVWRADTKSNVYGKNHDTGVSPVFAGGVEYAITPEIATRLEYQ

WTNNIGDAHTIGTRPDNGGVDKLMNIQIGELAKRTACPVVTIRFYEQEGLLPPPGRSRGNFRLY

PbrR 145 a.a.

GEEHVERLQFIRHCRSLDMPLSDVRTLLSYRKRPDQDCGEVNMLLDEHIRQVESRIGALLELKHH

LVELREACSGARPAQSCGILQGLSDCVCDTRGTTAHPSD

The sequencing graph of pLAP:

T7 terminator primer was used as the sequencing primer, and the target sequence (885 bp) was highlighted in yellow.

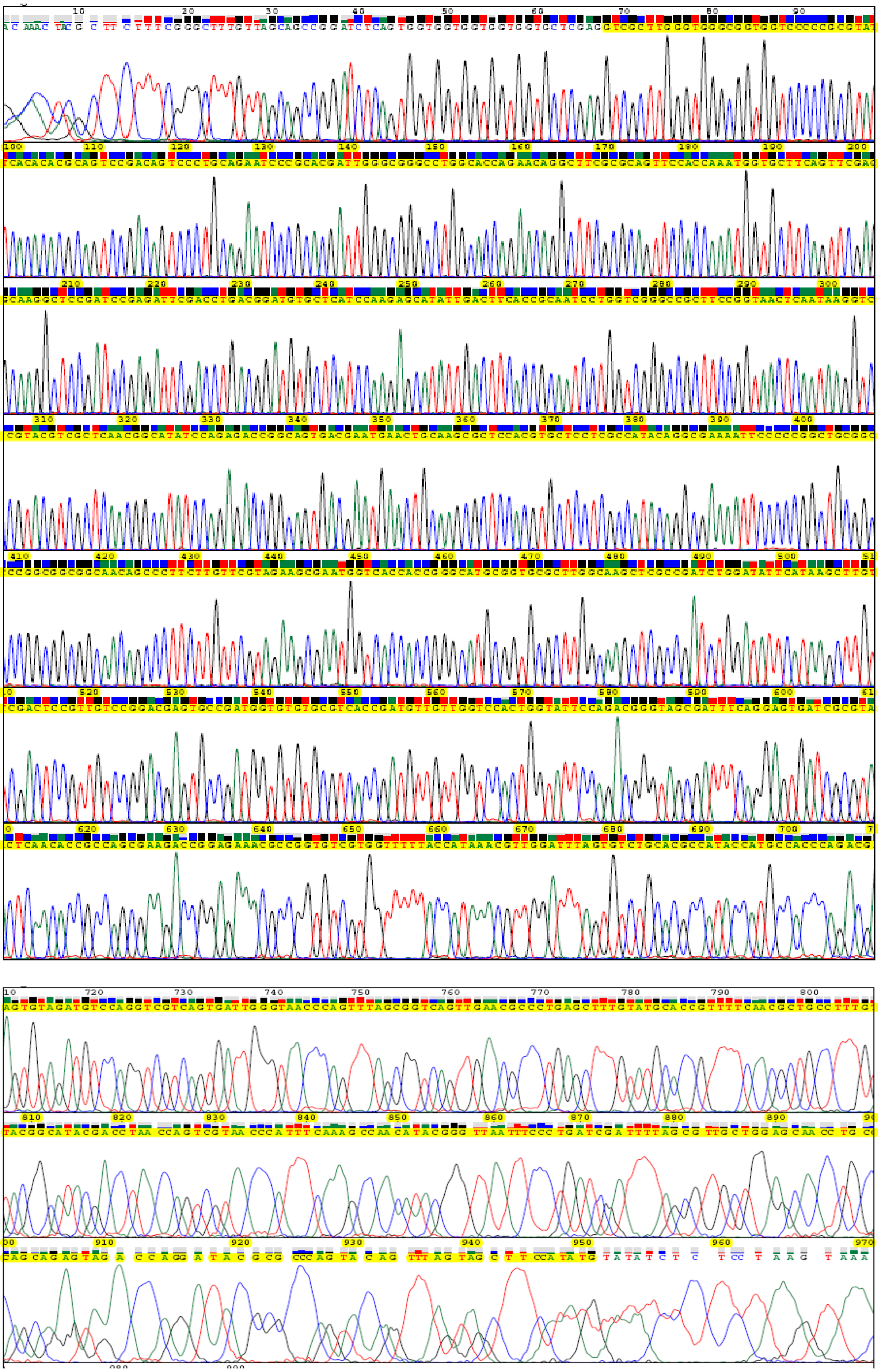

Figure S1. The codon optimized DNA sequence, deduced amino acid sequence, and sequencing graph of Lpp-OmpA (A) and Lpp-OmpA-PbrR (B).

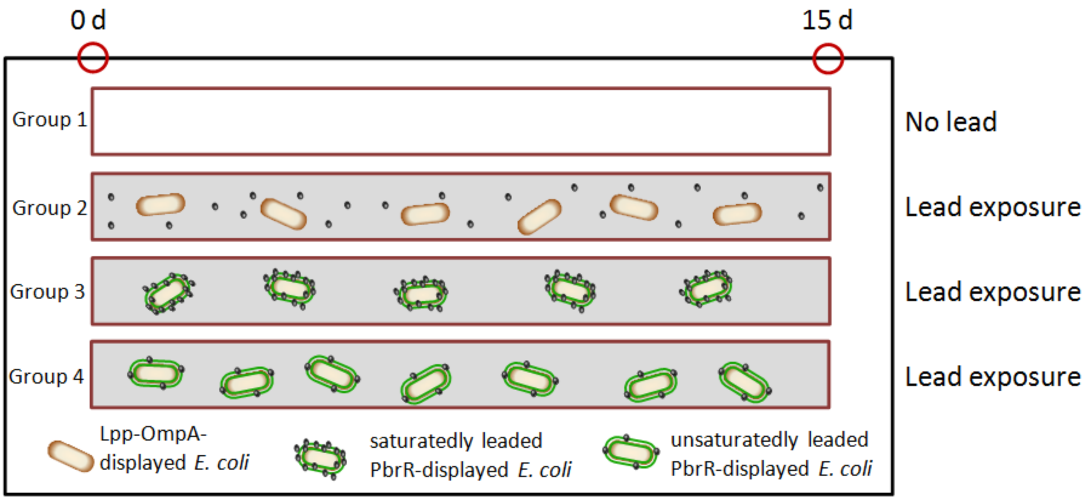

Figure S2. Animal experimental design. Thirty-six male KM mice were randomly divided into one control group of 8 mice (no lead exposed) and three other groups that were given different forms of lead (Group 2: lead acetate + Lpp-OmpA displayed *E. coli*; Group 3: saturatedly leaded PbrR-displayed *E. coli*; and Group 4: unsaturatedly leaded PbrR-displayed *E. coli*) using 20 μg lead/mouse daily. At 15 d, the lead levels in tissues were determined.

Supplemental Tables

Strains	Genotype/characteristics	References
Top10	F^- *mcr*A Δ(*mrr-hsd*RMS-*mcr*BC) φ80*lac*ZΔM15 Δ*lac*X74 *rec*A1 *ara*D139 Δ(*ara-leu*)7697 *gal*U *gal*K *rps*L (Str^R) *end*A1 *nup*G	Lab collection
BL21(DE3)pLysS	F^- *ompT* $hsdS_B$ (r_B^- m_B^-) *gal dcm* (DE3) pLysS ($Cm^{R)}$	Lab collection
**Plasmids**		
pUCm-T	TA cloning	Sangon Biotech
pT-*loa*	pUCm-T carrying *lpp-ompA*	This study
pT-*pbrR*	pUCm-T carrying *pbrR*	This study
pET-21a	Amp^r T7 promoter lac operator	Novagen
pLA	pET-21a expressing Lpp-OmpA under T7 promoter	This study
pLAP	pET-21a expressing Lpp-OmpA-PbrR fusion protein under T7 promoter	This study

Table S1. Bacterial strains and plasmids used in this study

**Primer**	**Sequence (5’ - 3’)**	**Restriction site**
Loa-F	GGATCTGCATATGAAAGCTACTAAACTG	*Nde* I
Loa-R	AGCGTCGACTCCGTTGTCCGG	*Sal* I
pbrR-F	CCCAAGCTTATGAATATCCAGATC	*Hin*d III
*pbr*R-R	CCGCTCGAGGTCGCTTGGGT	*Xho* I
T7 terminator primer	TGCTAGTTATTGCTCAGCGG	-

Table S2. Primers used for cloning and generating the synthetic gene encoding LOA-PbrR fusion protein. The restriction sites introduced in the primers are indicated on the right sides and their location within the primer sequence is underlined.
